# Notes on a Case of Alternating Oxaluria and Phosphaturia
^1^A paper read at a meeting of the Bristol Medico-Chirurgical Society on January 8th, 1913.


**Published:** 1913-03

**Authors:** F. H. Edgeworth

**Affiliations:** Physician to the Bristol Royal Infirmary, and Professor of Medicine, University of Bristol.


### NOTES ON A CASE OF ALTERNATING OXALURIA AND PHOSPHATURIA.[1]

BY

F. H. EDGEWORTH, M.D. Cantab., D.Sc. Lond.,

*Physician to the Bristol Royal Infirmary, and Professor of Medicine, University of Bristol.*

A FEW years ago there came under my care a case of alternating oxaluria and phosphaturia, the causation and treatment of which puzzled me for a long time and which finally admitted of a very simple solution.

The present state of our knowledge of these constituents of the urine is, shortly, as follows : calcium oxalate in the urine is, for the most part, derived from oxalates in the food, especially vegetables and fruit. These are mostly destroyed in the

[1] A paper read at a meeting of the Bristol Medico-Chirurgical Society on January 8th, 1913.

intestine, but a small proportion is absorbed, combines with calcium in the blood and lymph, and is excreted as calcium oxalate. The proportion absorbed is largely dependent on the acidity of the gastric contents. The calcium oxalate of the urine is normally in solution. It is generally thought that acid phosphate of sodium and magnesium salts are some of the factors which help to keep the calcium oxalate in solution, but this is doubtful; for instance, in the case recorded below crystals were only present when the urine became very acid, *i.e.* contained an excess of acid phosphate of sodium, and the administration of magnesium salts had no influence in preventing the occurrence of attacks.

Crystals of calcium oxalate may be present in the urine when it is passed. This may be due to ingestion of large quantities of oxalate-containing food-stuffs, *e.g.* cabbage, rhubarb, or it may be the result of gastric hyperacidity, or due to other causes of which we know little.

Such crystals may be the cause of hæmaturia or of renal colic or of both phenomena, quite apart from the existence of a calculus. It is curious that this—a matter of common knowledge—is not mentioned in many text-books.

Phosphates are deposited only in an alkaline urine—ammonium-magnesium phosphate in cases of ammoniacal fermentation; and earthy phosphates, either the crystalline "stellar" phosphate ($CaHPO_4.2H_2O$) or the amorphous phosphate ($Ca_3P_2O_8$) in cases where the alkalinity is caused by fixed alkalis. The two latter salts are rarely deposited until the urine cools, but exceptionally may be precipitated in the urinary passages, making the urine milky. Such amorphous phosphates rarely aggregate into masses, and hence do not tend to form calculi, and are generally quite unirritating, *e.g.* do not cause hæmaturia or renal colic: in this case, however, they did cause pain of a dull aching character.

The causes of alkalinity of the urine are but little known. One suggested cause is an increased excretion of calcium salts, brought about by a diminished excretion through the intestinal wall, from catarrh or other affection.

With these preliminary remarks, let me relate the case. Three years ago a man came under my care, suffering from right-sided renal colic and hæmaturia. He was 26 years of age, very strongly built, and had had no previous disease of any moment. The attack had set in quite suddenly, without known cause, and the colic was very severe, necessitating the use of morphia. Examination of the very red urine disclosed innumerable red-blood corpuscles and calcium oxalate crystals, but no epithelial casts; it was very acid in reaction. The colic lasted some hours, and then slowly subsided, leaving the right ureter sore and tender on palpation. The hæmaturia persisted for a day. A week or two later the phenomena recurred. The patient was then put on a milk diet, and as long as this was continued no attacks took place. But this could not be indefinitely continued, and a diet of meat, bread, and water was substituted, as one which contained a minimal quantity of oxalates.

But even on this diet attacks occasionally took place. It was found that the attacks were always heralded and accompanied by an increased acidity of the urine, and a dose of bicarbonate of sodium, taken directly the urine became more acid than usual, sometimes succeeded in checking the onset of an attack.

Another difficulty now occurred, from time to time—sometimes following an overdose of the alkali, sometimes, and more often, quite independently of any such dose—the urine became alkaline in reaction and milky in appearance when passed. This was found to be due to amorphous phosphates. No renal colic or hæmaturia occurred in such attacks, but pain did occur in the course of the right ureter, of a sore, aching character, very different from that attending the passage of oxalates.

The patient was thus between Scylla and Charybdis—if the urine became too acid, oxaluria with right renal colic with or without hæmaturia occurred; if alkaline, phosphaturia with pain in the right ureter was his lot.

This condition lasted many months, in spite of the administration of various drugs which have been recommended for these complaints.

At last the difficulty was solved. It was found that neither sort of attacks occurred if he ate slowly and carefully masticated his food, whereas they were liable to do so if he "bolted" it. A week or so later his diet of bread and meat was gradually changed to an ordinary one, still with the same result. During the last two years he has kept well, but for an occasional attack—invariably brought on by insufficient mastication—when his wife, for instance, did not come down to breakfast or went away on a visit.

For the whole of this time he has never had the slightest symptom of dyspepsia or of any intestinal affection.

The case, however, suggests that perhaps insufficient mastication may bring about gastric hyperacidity—though without any gastric symptoms. Possibly, too, it may cause some intestinal catarrh, or defect of intestinal absorption.

I have not found in books any statement of this as a cause of either oxaluria or phosphaturia. Watson remarked that frequently an over-acid state of the urine alternates with one of alkalescence, but he did not associate the condition with insufficient mastication of food, and no later authors so much as mention the subject. That it may be a cause is shown by the case related above, and its discovery gave a happy ending to the constantly occurring pain or discomfort to which he had been a martyr.

---

